# Draft genome sequence of *Enterococcus casseliflavus* strain MBBL_MP4 isolated from healthy bovine milk

**DOI:** 10.1128/mra.00865-26

**Published:** 2026-09-08

**Authors:** Kh. Yeashir Arafat, Md. Anamul Kabir Hemal, Md. Abu Ahsan Gilman, Md. Morshedur Rahman, Md. Robiul Karim, Ziban Chandra Das, Tofazzal Islam, M. Nazmul Hoque

**Affiliations:** 1 Molecular Biology and Bioinformatics Laboratory, Department of Gynecology, Obstetrics and Reproductive Health, Gazipur Agricultural University 198780, Gazipur, Bangladesh; 2 Department of Medicine, Gazipur Agricultural University 198780, Gazipur, Bangladesh; 3 Institute of Biotechnology and Genetic Engineering (IBGE), Gazipur, Bangladesh; Montana State University, Bozeman, Montana, USA

**Keywords:** *Enterococcus casseliflavus*, low pathogenicity, bovine milk, genome sequencing

## Abstract

We report the draft genome sequence of *Enterococcus casseliflavus* MBBL_MP4, recovered from healthy bovine milk. The 3.45-Mbp genome assembly comprises 27 contigs and indicates low pathogenic potential, with no acquired antimicrobial resistance or known virulence genes. This genome provides a valuable resource for the genomic characterization of bovine-associated *E. casseliflavus*.

## ANNOUNCEMENT

*Enterococcus casseliflavus* is a gram-positive, motile lactic acid bacterium commonly found in soil, plants, and the gastrointestinal tracts of animals (1). Although it is recognized as an opportunistic pathogen associated with infections such as bovine mastitis, certain strains possess beneficial characteristics, including antimicrobial activity and probiotic potential (2, 3). Here, we report the draft genome sequence of *E. casseliflavus* strain MBBL_MP4, isolated from healthy bovine milk collected from a dairy farm in Gazipur, Bangladesh (24.09582° N, 90.41252° E).

Milk samples were collected in November 2025 from clinically healthy dairy cows. The absence of mastitis was confirmed using the California Mastitis Test (4, 5) prior to sampling for bacterial isolation. Following collection, milk samples were transported to the laboratory in an ice box maintained at 4°C for immediate processing. Briefly, 100 µL of each milk sample was enriched in 5 mL of nutrient broth (HiMedia, India) and incubated overnight at 37°C (6, 7). Subsequently, 2 µL of the enriched culture was streaked onto nutrient agar (HiMedia, India) and incubated at 37°C for 24 h. A single colony was then subcultured on tryptic soy agar (Oxoid Ltd., Thermo Fisher Scientific, Hampshire, UK) and incubated overnight at 37°C under aerobic conditions (8). Presumptive *Enterococcus casseliflavus* isolates were further cultured on Columbia agar supplemented with 5% sheep blood (9). Species-level identification was confirmed using the VITEK 2 system (v9.01) (10).

Genomic DNA was extracted using QIAamp DNA Mini Kit (QIAGEN, Germany). Libraries were prepared with 1 ng DNA using the Nextera DNA Flex Kit (Illumina, USA) (11), and whole-genome sequencing was performed using the Illumina MiSeq platform (2 × 250 bp) (12). The quality of raw reads was examined with FastQC v0.11.3 (13), and low-quality reads were trimmed with Trimmomatic v0.39.2 (14). High-quality (Phred score > 30) reads were assembled using SPAdes v4.2.0 (15) and were annotated using the National Center for Biotechnology Information (NCBI) Prokaryotic Genome Annotation Pipeline v6.10 (16). Following initial identification with the VITEK 2 system, the taxonomic identity of strain MBBL_MP4 was confirmed by whole-genome sequence analysis using Kraken2 v2.1.3 (17). Completeness of genomes was estimated using CheckM v1.2.3 (18). Antimicrobial resistance genes (ARGs) were annotated by the CARD v3.2.4 (19), and virulence factor genes (VFGs) were identified using VFDB v6.0 (20) implemented in the ABRicate package (https://github.com/tseemann/abricate). The distribution of subsystems was analyzed using RAST v2.0 server (21) and pathogenicity risk to humans determined using PathogenFinder v1.1 (22). All tools were used with default parameters, unless otherwise specified.

The genomic characteristics of *E. casseliflavus* strain MBBL_MP4 are summarized in Table 1. The draft genome comprised 3,446,833 bp assembled into 27 contigs, with an average sequencing depth of 106.5 × and a GC content of 43.0%. Genome annotation identified 3,298 genes, including 3,232 protein-coding sequences (CDSs). No acquired ARGs and no known VFGs were detected in the genome of *E. casseliflavus* MBBL_MP4. In addition, the genome exhibited a low predicted pathogenicity score (0.407), indicating limited pathogenic potential (23, 24) and, together with the absence of detectable ARGs and VFGs, suggesting that this strain may be a promising candidate for future evaluation of probiotic-associated traits.

**TABLE 1:** Genomic features of *Enterococcus casseliflavus* MBBL_MP4 isolated from healthy bovine milk

Genomic features	*E. casseliflavus* MBBL_MP4
GenBank accession	JBSCTY000000000
BioSample accession	SAMN53123441
Assembled genome size (bp)	3,446,833
Number of paired-end raw reads	729,416
GC %	43
Coverage (×)	106.5×
N_50_ value (bp)	544,354
Number of contigs	27
Genome completeness	97.94%
Genome contamination	2.03%
Genes (total)	3,298
CDSs (total)	3,232
Genes (coding)	3,191
CDSs (with protein)	3,191
Genes (RNA)	66
Ribosomal RNAs	4, 2, 1 (5S, 16S, 23S)
Complete ribosomal RNAs	4, 1, 1 (5S, 16S, 23S)
Partial ribosomal RNAs	1 (16S)
Transfer RNAs	55
Non-coding RNAs	4
Pseudogenes (total)	41
CDSs (without protein)	41
Pseudogenes (ambiguous residues)	0 of 41
Pseudogenes (frameshifted)	8 of 41
Pseudogenes (incomplete)	29 of 41
Pseudogenes (internal stop)	9 of 41
Pseudogenes (multiple problems)	4 of 41
ARG	0
VFG	0
Number of subsystems	249
Pathogenicity score	0.407
Input proteome coverage (%)	0.25

## Data Availability

The whole-genome project of the *E. casseliflavus* MBBL_MP4 has been deposited in GenBank and the NCBI Sequence Read Archive SRR35976012 under BioProject accession number PRJNA1358723. The versions described in this paper are version JBSCTX000000000.1.
